# Sixteenth Annual Meeting of the American Society of Dental Surgeons

**Published:** 1856-10

**Authors:** Charles Bonsall


### Proceedings of Societies.

Dear Doctor.—Believing that some of your readers will feel interested in the closing proceedings of the American Society, I furnish you with a copy of the minutes.

Yours sincerely, CHARLES BONSALL.

The Sixteenth Annual Meeting of the American Society of Dental Surgeons convened according to adjournment at the Astor House, in New York, at 10 o'clock of August 5th, 1856. The following members were present, viz: Drs. Townsend, Dunning, Taylor, Allen, J. S. Clark, and Bonsall; no other members appearing and there being no quorum, they adjourned to meet at 3½ P. M., at the same place.

AFTERNOON SESSION.

Met according to adjournment. Present, Drs. Townsend, Dunning, Foster, Taylor, Clark and Bonsall. There not being still a quorum, the Secretary was requested to notify all members who could be reached in time, that a meeting would be held on Thursday morning, at 9 o'clock, at Hope Chapel, No. 718 Broadway, for the purpose of deciding finally as to the dissolution of the Society. Adjourned, as above.

THURSDAY MORNING, Aug. 7th.

Met according to adjournment at Hope Chapel, the following members being present, viz.: Drs. E. Townsend, J. Parmley, E. J. Dunning, R. Arthur, J. Allen, S. P. Miller, J. Taylor, R. P. Field, and C. Bonsall. The Secretary reported having notified by mail, as requested, some twenty members, in addition to those present on Tuesday, asking their attendance at this meeting.

The minutes of last year and Tuesday having been first read, they were adopted.

The Committee appointed last year being called upon, reported as follows:

MR. PRESIDENT AND GENTLEMEN.—Your Committee to whom was referred the subject of the propriety of dissolving the Society heretofore in existence as the American Society of Dental Surgeons, would respectfully report, that having given the subject that consideration which its importance demanded, they agree to offer the following resolution:

*Resolved*—That we deem it expedient to dissolve this Association, and that it be and is hereby adjourned *sine die.*

ELISHA TOWNSEND, *Chairman.*
E. J. DUNNING,
JNO. S. CLARK,
R. ARTHUR.

Which report was accepted, and on motion, Drs. Bonsall and Dunning were appointed a Committee to examine the Treasurer's books, and also to distribute the funds of the Society according to the constitution; after which the report of the Committee was unanimously adopted, and the Society adjourned *sine die.*

CHARLES BONSALL,
*Recording Sec'y.*

